# Fragment contribution models for predicting skin permeability using HuskinDB

**DOI:** 10.1038/s41597-023-02711-0

**Published:** 2023-11-23

**Authors:** Laura J. Waters, David J. Cooke, Xin Ling Quah

**Affiliations:** School of Applied Sciences, University of Huddersfield, Queensgate, Huddersfield, HD1 3DH UK

**Keywords:** Biophysical chemistry, Bioanalytical chemistry

## Abstract

Mathematical models to predict skin permeation tend to be based on animal derived experimental data as well as knowing physicochemical properties of the compound under investigation, such as molecular volume, polarity and lipophilicity. This paper presents a strikingly contrasting model to predict permeability, formed entirely from simple chemical fragment (functional group) data and a recently released, freely accessible human (i.e. non-animal) skin permeation database, known as the ‘Human Skin Database – HuskinDB’. Data from within the database allowed development of several fragment-based models, each including a calculable effect for all of the most commonly encountered functional groups present in compounds within the database. The developed models can be applied to predict human skin permeability (log*K*_p_) for any compound containing one or more of the functional groups analysed from the dataset with no need to know any other physicochemical properties, solely the type and number of each functional group within the chemical structure itself. This approach simplifies mathematical prediction of permeability for compounds with similar properties to those used in this study.

## Introduction

The rate and extent of permeation through human skin is a fundamental property that must be determined for any compound that may come into contact with skin, including a plethora of chemicals found in cosmetics and pharmaceutical products. In some cases this permeation may be desirable, such as transdermal drug delivery systems^1^, yet in other cases it should be avoided, such as for cosmetics and sun protection products^2^. Experimental determination of permeation through human skin is a complex and expensive process with alternatives frequently used such as animal skin^3^, even though these are known to often be unreliable predictive systems and bring their own issues regarding storage, preparation and predictive ability^4^. Thirty years ago, as an alternative to experimental determination, researchers began to consider mathematical models for predicting skin permeability including the well-known ‘Potts and Guy’ model^5^. In more recent years *in silico*-based systems have become more widespread comprising a range of computational approaches^6–8^, such as machine learning methods^9^ and quantitative structure-permeability relationships (QSPRs) that relate skin permeability to physicochemical properties and structural descriptors^10,11^. The vast majority of these models have relied on properties for a compound that are either well-known, for example molecular weight^12^, along with properties that can be predicted, for example lipophilicity and polar surface area^13^. Therefore, for a potentially permeable compound such models require a range of information to be known which may not be available. Very few studies have thought to try and simplify the structure-permeability relationship by quantifying group contributions from functional groups within the compound as a correlatable property. One study partially analysed functional group effects on the permeability of hydrocortisone esters from the perspective of their free energy of transfer of solute into the rate-limiting barrier of the stratum corneum but did not expand the relationship beyond this group of compounds^14^. A second study by the same authors analysed the functional group effects on permeability of methyl-substituted p-creosols yet again, did not expand the concept beyond this series of compounds^15^. In another study a range of properties were considered for predicting permeability including the number of carbon atoms present and some functional groups, although the latter were then mainly excluded as not significant within the dataset used, possibly as the dataset was comparatively small (n = 91) and based on animal (as opposed to human) skin data^16^.

In other analytical *in silico* scenarios fragment-based approaches have been used to predict useful information, for example for molecular property prediction^17^, and more relevant to this study, to predict permeation through the blood-brain barrier (BBB)^18^ whereby the models developed were noted as useful for the identification, selection and design of new drug candidates. A variety of additional uses of the group contribution approach can also be found in the literature, for example to predict the properties of aerosols^19^ and the well-established estimation of partition coefficients^20,21^. To be able to create such a model requires a comprehensive dataset of experimental data which may include information on experimental uncertainty with chemical descriptors^22^. For this study a freely accessible and comprehensive dataset of skin permeation data was used that solely comprised of human skin data with incorporated experimental parameters for each value included, known as HuskinDB^23^, making it the most relevant dataset available for consideration. Previous work from our group has established several models for predicting skin permeability using this dataset^24^ yet the models required knowledge of the physicochemical properties of the compound under investigation, such as partition coefficient (logP), topological surface area (TPSA) and molecular volume (MV). This study negates the need for such information by correlating skin permeation with only the knowledge of the type and number of functional groups present within the molecule in question, thus simplifying the predictive process immensely.

## Results

Firstly, a QSPR model was initially created using permeability coefficient (log*K*_p_) values from the HuskinDB dataset for 180 compounds containing ten functional groups (as listed in the Methods section) to confirm the validity of the concept. This dataset was selected from the original full dataset to avoid seven ‘unusual’ functional groups (boron, cyanide, epoxide, fluorine, nitro, phosphate and thiol) where only a small number of compounds included these groups and it was deemed insufficient data to create a reliable contribution for those particular groups. Where more than one log*K*_p_ value was available for a compound a series of experimental parameters were chosen to reduce the value to one, selected to most closely reflect those experienced *in vivo*, namely: abdomen source, epidermis and dermis layers, concentrated solute and an experimental donor solution temperature 31–35 °C. An experimental donor pH between 7 and 7.5 was selected to maximise the dataset as the majority of compounds that had specified pH only had data in this pH range available. The ten most commonly-encountered functional groups within the dataset (amide, amine, aromatic, bromine, carboxylic acid, chlorine, ester, ether, hydroxyl and ketone) were independently correlated with log*K*_p_ for each of the training set compounds (*n* = 144) to produce an equation (Eq. 1) that considers the individual contributions of each fragment to the overall permeation value. Coefficients of determination (*R*^2^) and root mean square error (RMSE) values for the training set (*n* = 144) and subsequent test set (*n* = 36) are presented along with Eq. 1 which displays the contribution for each functional group analysed. It should be noted that this equation also takes into consideration the prevalence of each functional group present in the molecule, for example if the compound contains two aromatic groups then the contribution value should be calculated as (+0.186 × 2).

1 $$\begin{array}{l}\begin{array}{l}\log Kp=-5.622+0.186(n\times {\rm{aromatic}})-0.369(n\times {\rm{amide}})-0.374(n\times {\rm{amine}})\\ \quad \quad \quad \;\,+\,0.329(n\times {\rm{bromine}})-0.757(n\times {\rm{carboxylic}}\;{\rm{acid}})+0.182(n\times {\rm{chlorine}})\\ \quad \quad \quad \;\,-\,0.272(n\times {\rm{ether}})-0.245(n\times {\rm{ester}})-0.349(n\times {\rm{hydroxyl}})-0.313(n\times {\rm{ketone}})\end{array}\\ {\rm{T}}{\rm{r}}{\rm{a}}{\rm{i}}{\rm{n}}{\rm{i}}{\rm{n}}{\rm{g}}\;{\rm{s}}{\rm{e}}{\rm{t}}:n=144,{R}^{2}=0.5002,{\rm{RMSE}}=0.76\\ {\rm{T}}{\rm{e}}{\rm{s}}{\rm{t}}\;{\rm{s}}{\rm{e}}{\rm{t}}:n=36,{R}^{2}=0.4003,{\rm{RMSE}}=0.96\end{array}$$

Although the resultant equation allows prediction for permeation for the first time using group contributions for any compound that contains one or more of the ten functional groups included, and the training and test sets both produced a reasonable correlation it is not as high as some models using physicochemical properties seen by others, such as that of Moss and Cronin’s analysis of steroids (*n* = 116, *R*^2^ = 0.82)^25^ or Magnusson *et al*. with an equation based on molecular weight alone (*n* = 87, *R*^2^ = 0.847)^26^.

For this reason, a second model was established to understand the relationship between predictive ability and experimental conditions. To achieve this, permeation data was divided into four experimental categories: skin source (breast/abdomen/thigh), skin type (epidermis/epidermis + dermis/dermis/stratum corneum), donor concentration (dilute/saturated) and experimental temperature (20–25/26–30/31–35/36–40 °C). A summary of the resultant equations with functional group contributions is displayed in Table 1.

**Table 1:** QSPR models for skin permeability (log*K*_p_) prediction using data extracted from HuskinDB based upon the ten most commonly encountered functional groups within the compounds analysed.

Skin Source	Skin Type	Donor Conc.	Exp. Temp. (°C)	No. of cmpds	R^2^	Equation
Breast	Epidermis	Diluted	36–40	9	0.9639	Log*K*p = −8.572 + 0.399 (Aromatic) + 2.426 (Ester) - 0.474 (Ether) - 2.071 (Hydroxyl)
Breast	Epidermis + Dermis	Saturated	36–40	6	0.7874	Log*K*p = −6.618 + 0.217 (Bromine) + 0.123 (Chlorine)
Breast	Epidermis + Dermis	Diluted	20–25	4	0.9703	Log*K*p = −4.226 - 0.040 (Chlorine)
Breast	Epidermis + Dermis	Diluted	31–35	20	0.5810	Log*K*p = −6.878 - 0.739 (Aromatic) + 0.548 (Amide) + 0.651 (Amine) + 1.451 (Carboxylic acid) - 0.325 (Ether) + 1.030 (Hydroxyl) - 1.037 (Ketone)
Breast	Epidermis + Dermis	Diluted	36–40	5	1.0000	Log*K*p = −5.243 - 0.253 (Chlorine) + 0.441 (Ester) + 0 (Ether) - 1.187 (Hydroxyl) + 0.826 (Ketone)
Abdomen	Epidermis	Saturated	20–25	10	N/A	Log*K*p = −7.850
Abdomen	Epidermis	Saturated	26–30	8	0.6325	Log*K*p = −5.645 - 0.046 (Ester) - 0.958 (Ether)
Abdomen	Epidermis	Diluted	20–25	36	0.6757	Log*K*p = −4.840 + 0.392 (Aromatic) - 1.654 (Amide) - 0.095 (Amine) + 0.424 (Bromine) + 0.266 (Chlorine) - 0.173 (Ester) - 0.975 (Hydroxyl) - 0.243 (Ketone)
Abdomen	Epidermis	Diluted	26–30	2	N/A	Log*K*p = −6.351
Abdomen	Epidermis	Diluted	31–35	36	0.7466	Log*K*p = −4.925 + 0.245 (Aromatic) - 0.054 (Amide) - 1.184 (Amine) - 1.582 (Carboxylic acid) + 0.589 (Chlorine) + 0.089 (Ester) + 0.537 (Ether) - 0.509 (Hydroxyl) - 0.019 (Ketone)
Abdomen	Epidermis	Diluted	36–40	43	0.5094	Log*K*p = −6.618 + 0.484 (Aromatic) + 0.966 (Amide) - 0.041 (Amine) - 0.252 (Carboxylic acid) + 0.850 (Chlorine) - 0.010 (Ester) - 0.665 (Ether) - 0.197 (Hydroxyl) −1.195 (Ketone)
Abdomen	Dermis	Saturated	20–25	8	N/A	Log*K*p = −6.674
Abdomen	Dermis	Diluted	20–25	16	0.8849	Log*K*p = −4.625 + 1.796 (Ester) + 0.021 (Ether) - 0.291 (Hydroxyl) - 1.542 (Ketone)
Abdomen	Dermis	Diluted	31–35	6	0.4186	Log*K*p = −5.528 - 0.283 (Aromatic)
Abdomen	Epidermis + Dermis	Saturated	26–30	4	N/A	Log*K*p = −8.632
Abdomen	Epidermis + Dermis	Diluted	20–25	4	N/A	Log*K*p = −6.063 (Hydroxyl)
Abdomen	Epidermis + Dermis	Diluted	26–30	8	0.8446	Log*K*p = −5.672 + 0.093 (Aromatic) - 0.660 (Amide) + 0.155 (Amine) + 0.422 (Carboxylic acid) - 0.603 (Hydroxyl)
Abdomen	Epidermis + Dermis	Diluted	31–35	45	0.2794	LogKp = −6.271 - 0.529 (Aromatic) - 0.148 (Amide) - 0.176 (Amine) + 1.136 (Carboxylic acid) + 0.221 (Chlorine) - 2.561 (Ester) + 0.426 (Ether) - 0.203 (Hydroxyl) - 0.665 (Ketone)
Abdomen	Epidermis + Dermis	Diluted	36–40	14	0.9661	Log*K*p = −5.007 + 0.478 (Ester) - 1.423 (Hydroxyl) + 0.925 (Ketone)
Abdomen	Stratum corneum	Diluted	26–30	3	0.2500	Log*K*p = −5.562 - 0.395 (Amide)
Abdomen	Stratum corneum	Diluted	31–35	3	1.0000	Log*K*p = −6.201 - 0.687 (Amide) + 0.357 (Hydroxyl)
Thigh	Epidermis	Diluted	31–35	3	1.0000	Log*K*p = −8.667 + 0.394 (Aromatic) + 0.488 (Amide)
Thigh	Epidermis	Diluted	36–40	3	1.0000	Log*K*p = −5.503 + 0.129 (Ether) - 1.989 (Hydroxyl)
Thigh	Epidermis + Dermis	Diluted	20–25	5	N/A	Log*K*p = −4.896
Thigh	Epidermis + Dermis	Diluted	26–30	17	0.8838	Log*K*p = −5.260 - 0.080 (Aromatic) + 0.240 (Amide) - 0.376 (Amine) - 0.181 (Chlorine) - 2.674 (Ester) + 0.119 (Ether) - 0.245 (Hydroxyl)
Thigh	Epidermis + Dermis	Diluted	31–35	3	0.5127	Log*K*p = −7.490 + 0.097 (Hydroxyl)
Thigh	Epidermis + Dermis	Diluted	36–ss40	21	0.3755	Log*K*p = −3.997 - 0.094 (Aromatic) - 1.469 (Amine)) + 0.576 (Ester) - 1.052 (Hydroxyl)

Where a group has no contribution to the equation it has been excluded from the final equation listed.

From all of the potential models created in Table 1, the most suitable for use is that which has a comparatively high number of compounds within the dataset and yet also as high as possible R^2^. Combining these two aspects ensures the chosen model will have both wide applicability for a range of compounds and a good degree of correlation, i.e., good predictive ability. Based on the data in Table 1 the most suitable model to meet these criteria is that based on abdomen skin with only the epidermis permeated and in a diluted donor solution at 31–35 °C. Under these conditions the number of compounds and coefficient of determination are both comparatively reasonable (from within the ranges displayed in Table 1), thus this model was selected as the most suitable. As before, the total number of compounds was separated into a training set (to derive Eq. 2) with associated coefficients of determination and root mean square error (RMSE) values for both the training and subsequent test sets.

2 $$\begin{array}{l}\begin{array}{l}\log Kp=-4.916+0.168(n\times {\rm{aromatic}})-0.176(n\times {\rm{amide}})-1.143(n\times {\rm{amine}})\\ \,\quad \quad \;+\,0(n\times {\rm{bromine}})-1.521(n\times {\rm{carboxylic}}\;{\rm{acid}})+0.616(n\times {\rm{chlorine}})\\ \quad \quad \,\;+\,0.601(n\times {\rm{ether}})+0.145(n\times {\rm{ester}})-0.512(n\times {\rm{hydroxyl}})-0.131(n\times {\rm{ketone}})\end{array}\\ {\rm{T}}{\rm{r}}{\rm{a}}{\rm{i}}{\rm{n}}{\rm{i}}{\rm{n}}{\rm{g}}\;{\rm{s}}{\rm{e}}{\rm{t}}:n=29,{R}^{2}=0.7125,{\rm{RMSE}}=0.71\\ {\rm{T}}{\rm{e}}{\rm{s}}{\rm{t}}\;{\rm{s}}{\rm{e}}{\rm{t}}:n=7,{R}^{2}=0.8931,{\rm{RMSE}}=0.49\end{array}$$

**Fig. 1:**
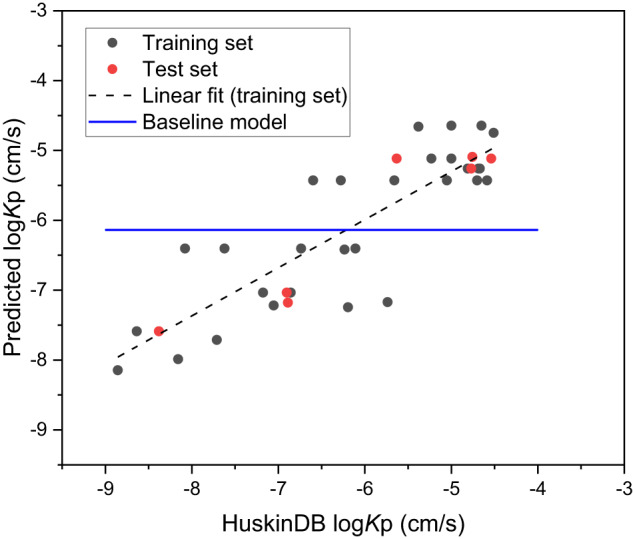
Predicted (from Eq. 2), experimental (HuskinDB) and baseline model log*K*_p_ values (cm/s) for the training and test sets.

This model could be simplified even further by removing the bromine contribution as with a value of zero it is unnecessary for inclusion. Figure 1 displays the relationship between the predicted and experimental log*K*_p_ values for the 36 compounds analysed using Eq. 2 based upon HuskinDB logarithmic *K*_p_ values expressed in cm/s.

## Discussion

As visualised in Fig. 1, there is a clear correlation between the predicted log*K*_p_ values and those found experimentally from HuskinDB, confirming the relationship between the functional groups present in a compound and their influence on permeation. Even though the dataset for Eq. 2 was far smaller than that for Eq. 1, Eq. 2 still included a range of compounds that included all of the functional groups under investigation. The *R*^2^ value obtained with the training set, and even more importantly the test set, indicate that this model is far superior to Eq. 1 from the far larger dataset. In comparison with our previously proposed model^24^ that utilised physicochemical data for each compound, the model presented in Eq. 2 can be considered superior (despite the smaller dataset) based upon the higher *R*^2^ values (0.7125 and 0.8931 vs. 0.5042 and 0.5057) and lower RMSE values (0.71 and 0.49 vs. 0.73 and 0.84) for the training and test sets respectively. Statistical significance using a two-tailed t-distribution for the training and test sets in Eq. 2 was further confirmed whereby ρ was calculated to be 8.7 × 10^−9^ (n = 29) and 1.3 × 10^−3^ (n = 7) respectively, i.e. far smaller than the standard accepted limit of 0.05.

As this is the first model of its kind (using functional groups to predict permeation) there are limitations in appropriate models for comparison. Figure 1 includes a baseline model produced using a combination of the permeation values extracted from HuskinDB and the average permeation value from all of the HuskinDB data used (−6.14), presented as a horizontal relationship compared with the far more linear relationships observed for the training and test datasets. To further corroborate the findings, RMSE values for both training and test datasets were calculated using mean baseline models. For the training set used in Eq. 1 (*n* = 144) and Eq. 2 (*n* = 29) the mean baseline models provided RMSE values of 1.07 and 1.31 respectively, far higher than those calculated from the equations themselves. This indicates that using the models will provide a better prediction of permeation compared with simply taking an average value from the dataset.

Furthermore, this model is also more suitable than those published by others, such as the well-known ‘Potts and Guy’ model^5^ (R^2^ = 0.67) or the United States Environmental Protection Agency DERMWIN™ model^27^ (R^2^ = 0.66), both based on partition coefficient and molecular weight data, as opposed to functional group data. The same set of compounds as those used in the training and test sets for Eq. 2 were then analysed using the ‘Potts and Guy’ model^5^ and the DERMWIN™ model (log Kp (cm/h) = −2.80 + 0.66 logKow - 0.0056 MW)^27^. For both models an *R*^2^ of 0.504 was calculated for the training sets and 0.737 and 0.738 for the test sets, i.e. all four values were lower than obtained for Eq. 2. Both models also exhibited higher RMSE values of 1.23 and 1.17 for the training sets with 1.15 and 1.10 for the test sets respectively, i.e. higher than obtained for Eq. 2. Even when compared alongside a far more complex QSAR model based on substructural molecular fragments that considers types of bonds (single/double/triple)^28^, our model performed well with a higher test set *R*^2^ value (0.893 vs. 0.630). Furthermore, the aforementioned publication does not specify the exact values of the separate contributions. For example, our ‘constant’ contribution in Eq. 2 is defined as −4.916 (similar to their value of approximately −5) yet their exact values for each fragment contribution are not provided. Their lack of inclusion of specific values does not facilitate the same level of usefulness for readers to facilitate permeation calculation that our approach provides.

**Fig. 2:**
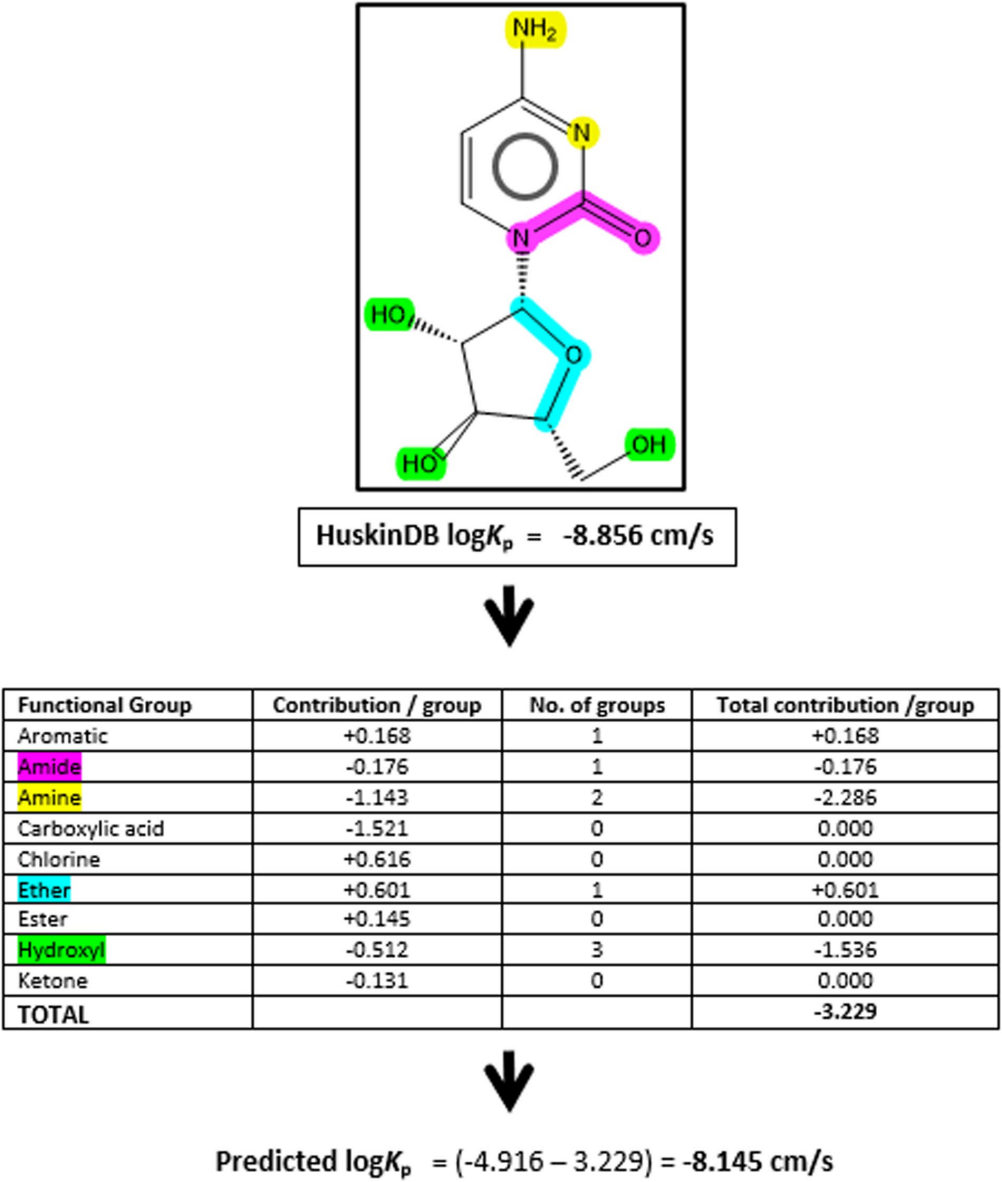
An example of how the mathematical model can be applied for a given compound using Eq. 2, illustrated using a compound from within HuskinDB (cytarabine) to highlight the correlation between the experimental and predicted values.

Therefore, we propose that our model could be used to predict permeation for any compound that contains one or more functional groups within the compound and no other physicochemical information is required. From a practical perspective, it is envisaged that the model can be applied very simply by a researcher once they have identified the chemical composition of their compound under investigation. From this point they can then use the model to calculate the overall contribution for the groups and insert that into the equation to achieve a predicted permeation value, as summarised for a model compound (cytarabine) in Fig. 2.

Using the model in this way transforms the theoretical concept to a practical and useful tool for researchers to use when wishing to predict permeation, i.e., taking a dataset, transforming it into a model and then confirming its suitability for predictive purposes. It could be argued that there are limits on the range of compounds that can be predicted from such equations, for example only for those with similar molecular mass or lipophilicity ranges to those in the dataset. At this time, it is not possible to confirm how far beyond the included range of properties the model would be reliable thus reasonable caution should be taken when extending beyond such limits. Furthermore, the authors acknowledge the limited size of the dataset (which can introduce stochastic effects), and that larger datasets would provide access to more sophisticated models such as random forests. In summary, this approach dramatically simplifies mathematical prediction whilst also ensuring the obtained values are human-relevant and therefore offers an exciting way forward for simple, yet precise, permeability prediction for a wide variety of compounds.

## Methods

All *K*_p_ values (cm/s) analysed in the study were considered as log*K*_p_ values from within the HuskinDB database^29^, expressed as logarithmic (log*K*_p_) values as this is standard procedure. log*K*_p_ values were analysed with the ten most commonly encountered functional groups in the dataset: amide, amine, aromatic, bromine, carboxylic acid, chlorine, ester, ether, hydroxyl and ketone.

Our goal was to fit a multiple regression model of the form^30^:

$y={a}_{0}+{a}_{1}{x}_{1}+{a}_{2}{x}_{2}+{a}_{3}{x}_{3}\ldots$ or $y={a}_{0}+{\sum }_{j=1}^{n}\left({a}_{j}{x}_{j}\right)$ to the data available,where *a*_0_ is the best estimate of log*K*_p_ if no information about the functional groups is present or they have no effect (equivalent to the intercept of a simple regression model) and *a*_j_ are the parameters relating to each property, the amount that is added or subtracted to estimate log*K*_p_ due to the presence of each functional group of type *j* present in the molecule of interest. *y* is log*K*_p_ for the molecule and *x*_j_ the number of functional groups *j* present in the molecule.

This is done by minimising the quantity *q*, which is the sum of the deviation between observed and predicted values of log*K*_p_ squared:

$$q=\mathop{\sum }\limits_{i=1}^{m}{\left({y}_{i}-\left({a}_{0}+\mathop{\sum }\limits_{j=1}^{n}{a}_{j}{x}_{ij}\right)\right)}^{2}$$

It can be shown that the values of *a*_j_ that optimise this expression, when expressed in matrix form, are:

$${\boldsymbol{a}}={\left({{\boldsymbol{X}}}^{{\boldsymbol{T}}}.{\boldsymbol{X}}\right)}^{-1}\left({{\boldsymbol{X}}}^{{\boldsymbol{T}}}.{\boldsymbol{Y}}\right)$$

where ***a*** is a column vector containing the *n* + 1 fitted parameters *a*_0_, *a*_1_*, …. a*_n_

***Y*** is a column vector containing the *m* observed values of log*K*_p_

***X*** is matrix with m rows and n + 1 columns. Each row containing the number of each of the *n* functional groups present, with the first column being filled with 1’s as there is no data associated with the *a*_0_ term (it is 1 *a*_0_, rather than *x*_0_ *a*_0_).

***X***^**T**^ is the same matrix transposed so it has m columns and *n* + 1 rows. This is required, to allow the matrices to be multiplied.

Once the estimates for the parameters *a*_0_, *a*_1_ … had been determined a method was required to test the goodness of fit, whether the parameter is statistically different to it being zero or whether incorporating a term associated with a specific functional group gives anything significant to the model. This is done, in two ways. Firstly, an ANOVA table was constructed which tests the hypothesis that all the fitted values are equal to zero by calculating the *F* statistic and its associated probability. Using the same matrix notation as above, it can be shown that the maximum likelihood estimate for the standard deviation in the fitted values of *y* is:

$$\widehat{{\rm{\sigma }}}=\sqrt{\frac{{{\boldsymbol{Y}}}^{{\bf{T}}}{\boldsymbol{Y}}-{{\boldsymbol{a}}}^{{\bf{T}}}{{\boldsymbol{X}}}^{{\bf{T}}}{\boldsymbol{Y}}}{m}}$$

and the fitted parameter is considered not significantly different to zero if:

$$\left|{a}_{j}\right| < {t}_{m-n}.\widehat{{\rm{\sigma }}}\sqrt{\frac{m\left|{c}_{jj}\right|}{m-n}}$$

where *c*_jj_ is the *j*th diagonal element of the square matrix (***X***^**T**^.***X***)^−1^ used in fitting the regression parameters and *t*_m−n_ is the *t* statistic on *m*−*n* degrees of freedom at the required level of significance. This analytical approach seemed suitable as it has been previously applied to a wide variety of applications^31–35^. Having determined a method for fitting the regression parameters and then assessing their significance, the next task was to determine the most appropriate parameters to include in the model. For this we adopted the ‘top down’ approach whereby a regression model was fitted using all possible parameters and the least significant was then removed (or each parameter that is not significantly different to zero removed in turn) and a model with one fewer parameter fitted. This process was repeated until all the parameters included in the model were significantly different to zero at the required level.

Two approaches were adopted for creation of an optimised model using different subsets of data. Firstly, 180 compounds from the dataset were included for analysis (after removal of extreme outliers and unusual functional groups). Secondly, as with our previous study^24^, four experimental variables: skin source (breast/ abdomen/ thigh), skin layer used (epidermis/ dermis/ epidermis + dermis/ stratum corneum), concentration of donor solution (neat/diluted) and donor solution temperature (20–25/26–30/31–35/36–40 °C) were considered. As before, these four variables included a total of 96 scenarios yet only 27 were analysed (i.e., where *n* > 1), with 71 compounds from the 253 in total excluded as they did not fulfil the requirement to have at least one specified experimental variable. It should be noted that some of the remaining compounds were sometimes considered in more than one scenario where multiple log*K*_p_ values were provided under different experimental variables.

Multiple linear regression analysis (using Microsoft Excel (Data Analysis), Microsoft 365^®^) with the ten functional groups created models with their associated coefficients of determination (*R*^2^). Data was divided randomly into training (80%) and test (20%) sets using the training set to form an equation for each model which was then reviewed using the associated test set. The decision to use an 80:20 split was chosen to follow that used in our previous work, which itself was selected based on supporting literature^35^. For comparative analysis with existing models all calculated logP/logKow and MW values were extracted from (www.molinspiration.com^36^) for consistency.

### Supplementary information

Supp 1

## Data Availability

The authors declare that the data supporting the findings of this study are available within the paper. Literature data used in the paper was extracted from HuskinDB (drug-design.de) which has been presented in this journal as ‘HuskinDB, a database for skin permeation of xenobiotics’^23^.

## Code availability

No custom code was used to generate or process the data described in this manuscript.
