# Supplementary material for: Fragment contribution models for predicting skin permeability using HuskinDB
Source: Sci Data. 2023 Nov 23;10:821. doi: 10.1038/s41597-023-02711-0 (PMC10667307; doi:10.1038/s41597-023-02711-0)
Supplement: Supplementary file 1 — Supp 1 [file 41597_2023_2711_MOESM1_ESM.docx]

Compounds, associated HuskinDB, predicted and alternative model values

Training compounds for Equation 1

| Compound | HuskinDB Log*K*p (cm/s) | Predicted (Eqtn. 1) Log*K*p (cm/s) |
| --- | --- | --- |
| 1,1,1-Trichloropropanone | -5.176 | -5.389 |
| 1,1-Dichloropropanone | -4.923 | -5.571 |
| 1,2,4-Benzenetriol | -7.456 | -6.483 |
| 1-Butanol | -5.816 | -5.971 |
| 1-Decanol | -6.410 | -5.971 |
| 1-Hexanol | -5.778 | -5.971 |
| 1-Hexyl-2-Pyrrolidone | -5.208 | -5.991 |
| 1-Methoxy-2-Propanol | -6.397 | -6.243 |
| 1-Octyl-2-Azacycloheptanone | -4.836 | -5.991 |
| 1-Octyl-2-Pyrrolidone | -4.827 | -5.991 |
| 1-Pentanol | -5.778 | -5.971 |
| 1-Propanol | -6.556 | -5.971 |
| 2-(2-Ethoxyethoxy)ethanol | -7.436 | -6.515 |
| 2-(2-Methoxyethoxy)ethanol | -7.242 | -6.515 |
| 2,4-Dichlorophenol | -4.778 | -5.421 |
| 2-Butoxyethanol | -5.340 | -6.243 |
| 2-Ethoxyethyl acetate | -6.649 | -6.139 |
| 2-Hydroxypropyl Nicotinate | -8.025 | -6.404 |
| 2-Methoxyethanol | -6.095 | -6.243 |
| 2-Naphthol | -4.757 | -5.599 |
| 2-Phenylethanol | -5.276 | -5.785 |
| 2-Phenylphenol | -6.355 | -5.599 |
| 2-Propoxyethanol | -6.923 | -6.243 |
| 3,4-Xylenol | -5.000 | -5.785 |
| 4,4'-Methylenedianiline | -6.219 | -5.998 |
| 4-Chloro-m-phenylenediamine | -6.234 | -6.002 |
| 4-Hydroxybenzyl alcohol | -6.255 | -6.134 |
| 4-Hydroxyphenylacetamide | -6.903 | -6.154 |
| 4-Hydroxyphenylacetic acid | -6.158 | -6.542 |
| 5-Aminolevulinic acid | -7.711 | -7.066 |
| Acetic acid | -6.079 | -6.379 |
| Acetylsalicylic acid | -5.696 | -6.438 |
| Acyclovir | -7.079 | -7.736 |
| Aldosterone | -8.079 | -7.259 |
| Atenolol | -7.857 | -7.169 |
| Barbital | -7.509 | -6.673 |
| Benzene | -4.511 | -5.436 |
| Benzoic acid | -5.156 | -6.193 |
| Benzyl nicotinate | -5.348 | -5.869 |
| Bisoprolol Fumerate | -7.125 | -7.977 |
| Bromoacetic acid | -6.410 | -6.050 |
| Bromochloroacetic acid | -6.352 | -5.868 |
| Bromodichloromethane | -4.301 | -4.929 |
| Butan-2-one | -5.903 | -5.935 |
| Butobarbital | -7.268 | -6.673 |
| Butoxyethanol | -5.982 | -6.243 |
| Butyl nicotinate | -5.335 | -6.055 |
| Caffeine | -6.828 | -6.736 |
| Carvacrol | -4.810 | -5.785 |
| Catechol | -6.066 | -6.134 |
| Chlorocresol | -5.000 | -5.603 |
| Chlorodibromomethane | -4.255 | -4.782 |
| Chloroform | -4.352 | -5.076 |
| Chloroxylenol | -4.651 | -5.603 |
| Chlorpheniramine | -6.491 | -5.816 |
| Codeine | -7.866 | -6.703 |
| Cortexolone | -7.681 | -6.946 |
| Cortexone | -6.903 | -6.597 |
| Corticosterone | -7.556 | -6.946 |
| Cortisone | -7.380 | -7.259 |
| Dibromoacetic acid | -6.141 | -5.721 |
| Dichloroacetic acid | -6.278 | -6.015 |
| Diclofenac | -6.556 | -6.017 |
| Diethyl ether | -5.352 | -5.894 |
| Diethylcarbamazine | -7.459 | -6.739 |
| Diethylene glycol mono n-butyl ether acetate | -7.778 | -6.411 |
| Diethylene glycol monobutyl ether | -8.004 | -6.515 |
| Dipropylene glycol mono methyl ether | -7.515 | -6.515 |
| Estradiol | -5.829 | -6.134 |
| Estriol | -7.954 | -6.483 |
| Ethacrynic acid | -7.380 | -6.414 |
| Ethanol | -6.352 | -5.971 |
| Ethyl 3-ethoxypropionate | -6.669 | -6.139 |
| Ethyl nicotinate | -5.755 | -6.055 |
| Ethyl p-aminobenzoate | -5.255 | -6.055 |
| Ethylene glycol mono isopropyl ether | -7.125 | -6.243 |
| Ethylene glycol mono methyl ether acetate | -6.637 | -6.411 |
| Etodolac | -5.683 | -6.653 |
| Etorphine | -6.000 | -7.052 |
| Fentanyl | -5.556 | -5.993 |
| Histidine | -7.812 | -7.315 |
| Hydrocortisone 21-(6-hydroxyhexanoate) | -6.597 | -7.540 |
| Hydrocortisone 21-(N,N-dimethylsuccinamate) | -7.730 | -7.560 |
| Hydrocortisone 21-Hemisuccinate | -6.757 | -7.191 |
| Hydrocortisone 21-methylsuccinate | -7.234 | -7.436 |
| Hydrocortisone 21-pimelamate | -6.607 | -7.560 |
| Hydrocortisone 21-propionate | -6.038 | -7.191 |
| Hydrocortisone 21-succinamate | -8.141 | -7.560 |
| Hydroquinone | -6.506 | -6.134 |
| Hydroxypregnenolone | -6.778 | -6.633 |
| Ibuprofen mannoside | -6.548 | -7.349 |
| Indomethacin | -6.997 | -6.280 |
| Isoquinoline | -5.332 | -5.624 |
| Ketoprofen | -6.768 | -6.320 |
| Ketoprofen mannoside | -7.594 | -7.163 |
| Ketorolac | -5.799 | -6.694 |
| Lidocaine | -5.707 | -5.805 |
| Linolenic acid | -5.988 | -6.379 |
| Mannitol | -7.923 | -7.716 |
| m-Cresol | -5.373 | -5.785 |
| Meperidine | -5.988 | -6.055 |
| Methanol | -5.539 | -5.971 |
| Methyl 4-hydroxybenzoate | -5.632 | -6.030 |
| Methyl p-aminobenzoate | -5.195 | -6.055 |
| Methyl salicylate | -5.000 | -6.030 |
| Methyltriglycol nicotinate | -7.308 | -6.871 |
| Metoprolol | -6.637 | -6.703 |
| N4-acetyl-4-amino-1-[(2R, 3S, 4R, 5R)-3-4-dihydroxy-5(hydroxymethyl)oxolan-2-yl] pyrimidin-2-one | -6.889 | -7.867 |
| Naphthol | -5.141 | -5.599 |
| Naproxen | -6.096 | -6.279 |
| Naproxen glucoside | -7.886 | -7.435 |
| n-Hexyl nicotinate | -5.303 | -6.055 |
| Nicotine | -5.546 | -6.184 |
| n-Nitrosodiethanolamine | -5.944 | -6.694 |
| Nortriptyline hydrochloride | -6.444 | -5.442 |
| Octylparaben | -5.588 | -6.030 |
| Oleic acid | -6.442 | -6.379 |
| o-Phenylenediamine | -6.903 | -6.184 |
| o-t-Butylphenol | -4.685 | -5.785 |
| Ouabain | -9.653 | -9.203 |
| Oxprenolol | -6.369 | -6.703 |
| Paraquat | -8.617 | -5.998 |
| p-Ethylphenol | -5.014 | -5.785 |
| Phenobarbital | -6.900 | -6.487 |
| Phenol | -5.586 | -5.785 |
| Phloroglucinol | -6.066 | -6.483 |
| p-n-Butylphenol | -4.671 | -5.785 |
| p-Phenylenediamine | -7.176 | -6.184 |
| Prazosin hydrochloride | -5.711 | -7.563 |
| Prednisolone | -7.906 | -7.295 |
| Pregnenolone | -6.380 | -6.284 |
| Progesterone | -5.255 | -6.248 |
| Propoxur | -5.878 | -6.077 |
| Propranolol-HCl | -7.879 | -6.063 |
| Propylparaben | -5.410 | -6.030 |
| Resorcinol | -6.626 | -6.134 |
| Salicylic acid | -5.136 | -6.542 |
| Sucrose | -8.840 | -9.230 |
| Testosterone | -5.856 | -6.284 |
| Theophylline | -7.125 | -6.736 |
| Thymol | -4.770 | -5.785 |
| Trichloroacetic acid | -6.278 | -5.833 |
| Triglycol nicotinate | -8.561 | -6.948 |
| Water | -6.429 | -5.971 |

Test compounds for Equation 1

| Compound | HuskinDB Log*K*p (cm/s) | Predicted (Eqtn. 1) Log*K*p (cm/s) |
| --- | --- | --- |
| 17-Hydroxyprogesterone | -6.778 | -6.597 |
| 1-Octanol | -5.642 | -5.971 |
| 2,4,6-Trichlorophenol | -4.783 | -5.239 |
| 2-Ethoxyethanol | -7.687 | -6.243 |
| 2-Phenoxyethanol | -6.430 | -6.057 |
| 4-Propoxyphenol | -5.380 | -6.057 |
| Amobarbital | -6.201 | -6.673 |
| Aspartic acid | -7.585 | -7.510 |
| Celiprolol Hydrochloride | -6.785 | -7.305 |
| Chloral hydrate | -5.965 | -5.774 |
| Chloroacetic acid | -6.515 | -6.197 |
| Digitoxin | -8.442 | -9.244 |
| Dimethylethylamine | -6.079 | -5.996 |
| Ephedrine | -6.326 | -6.159 |
| Estrone | -6.000 | -6.098 |
| Hydrocortisone 21-Hemipimelate | -6.301 | -7.191 |
| Ibuprofen | -4.996 | -6.193 |
| Ibuprofen glucoside | -6.945 | -7.349 |
| Ketoprofen glucoside | -7.731 | -7.163 |
| L-alanyl-L-tryptophan | -8.380 | -6.750 |
| Lysine | -7.328 | -7.127 |
| Methotrexate | -6.840 | -9.565 |
| Methyl 4-hydroxyphenylacetate | -5.255 | -6.030 |
| Methyl nicotinate | -5.966 | -6.055 |
| Naproxen mannoside | -7.700 | -7.435 |
| o-Chlorophenol | -5.037 | -5.603 |
| o-Cresol | -5.360 | -5.785 |
| p-Bromophenol | -4.999 | -5.456 |
| p-Chlorophenol | -4.996 | -5.603 |
| p-Cresol | -5.313 | -5.785 |
| Propranolol | -6.306 | -5.896 |
| Pyrogallol | -6.366 | -6.483 |
| Raffinose | -8.208 | -10.821 |
| Triclosan | -7.269 | -5.325 |
| Trimethylamine | -7.284 | -5.996 |
| Urea | -7.317 | -5.991 |

Training compounds for Equation 2

| Compounds | HuskinDB LogKp (cm/s) | Predicted (Eqtn. 2) Log*K*p (cm/s) | ‘Potts and Guy’ model Log*K*p (cm/s) | DERMWIN model Log*K*p (cm/s) |
| --- | --- | --- | --- | --- |
| 1-Butanol | -5.659 | -5.428 | -5.957 | -6.032 |
| 1-Decanol | -4.701 | -5.428 | -4.319 | -4.504 |
| 1-Hexanol | -5.051 | -5.428 | -5.411 | -5.523 |
| 1-Octanol | -4.586 | -5.428 | -4.865 | -5.013 |
| 2-Amino-4-Nitrophenol | -8.079 | -6.403 | -6.466 | -6.500 |
| 2-Amino-4-Nitrophenol | -6.737 | -6.403 | -6.466 | -6.500 |
| 2-Nitro-p-Phenylenediamine | -6.857 | -7.034 | -6.964 | -6.963 |
| 4-Amino-2-Nitrophenol | -7.622 | -6.403 | -6.757 | -6.771 |
| 4-Amino-2-Nitrophenol | -6.109 | -6.403 | -6.757 | -6.771 |
| 4-Chloro-m-phenylenediamine | -6.234 | -6.418 | -6.389 | -6.429 |
| 4-Propoxyphenol | -5.380 | -4.659 | -5.531 | -5.631 |
| 5-Aminolevulinic acid | -7.711 | -7.711 | -8.470 | -8.364 |
| Benzene | -4.511 | -4.748 | -5.399 | -5.513 |
| Caffeine | -7.053 | -7.218 | -7.442 | -7.404 |
| Carvacrol | -4.810 | -5.260 | -4.511 | -4.683 |
| Chlorocresol | -5.000 | -4.644 | -5.068 | -5.201 |
| Chloroxylenol | -4.651 | -4.644 | -5.019 | -5.154 |
| Cytarabine | -8.856 | -8.145 | -9.154 | -8.992 |
| Ethanol | -6.278 | -5.428 | -6.538 | -6.575 |
| Flufenamic acid | -6.194 | -7.244 | -4.579 | -4.737 |
| L-alanyl-L-tryptophan | -8.637 | -7.588 | -9.144 | -8.980 |
| Mannitol | -8.158 | -7.988 | -9.612 | -9.422 |
| Methyl 4-hydroxybenzoate | -5.230 | -5.115 | -6.071 | -6.133 |
| Methyl salicylate | -5.000 | -5.115 | -5.716 | -5.803 |
| N4-hexanoyl-4-amino-1-[(2R, 3S, 4R, 5R)-3-4-dihydroxy-5(hydroxymethyl)oxolan-2-yl] pyrimidin-2-one | -5.737 | -7.170 | -11.342 | -11.019 |
| o-t-Butylphenol | -4.685 | -5.260 | -4.675 | -4.835 |
| p-n-Butylphenol | -4.671 | -5.260 | -4.859 | -5.006 |
| p-Phenylenediamine | -7.176 | -7.034 | -6.896 | -6.903 |
| Water | -6.597 | -5.428 | -6.616 | -6.649 |

Test compounds for Equation 2

| Compound | HuskinDB Log*K*p (cm/s) | Predicted (Eqtn. 2) Log*K*p (cm/s) | ‘Potts and Guy’ model Log*K*p (cm/s) | DERMWIN model Log*K*p (cm/s) |
| --- | --- | --- | --- | --- |
| 2-Naphthol | -4.757 | -5.092 | -5.305 | -5.421 |
| Butyl 4-hydroxybenzoate | -4.538 | -5.115 | -5.305 | -5.418 |
| L-alanyl-L-tryptophan | -8.380 | -7.588 | -9.144 | -8.980 |
| Methyl 4-hydroxybenzoate | -5.632 | -5.115 | -6.071 | -6.133 |
| N4-acetyl-4-amino-1-[(2R, 3S, 4R, 5R)-3-4-dihydroxy-5(hydroxymethyl)oxolan-2-yl] pyrimidin-2-one | -6.889 | -7.178 | -9.595 | -9.399 |
| o-Phenylenediamine | -6.903 | -7.034 | -6.349 | -6.394 |
| Thymol | -4.770 | -5.260 | -4.845 | -4.993 |
